# RNA and mRNA Nitration as a Novel Metabolic Link in Potato Immune Response to *Phytophthora infestans*

**DOI:** 10.3389/fpls.2018.00672

**Published:** 2018-05-29

**Authors:** Karolina Izbiańska, Jolanta Floryszak-Wieczorek, Joanna Gajewska, Barbara Meller, Daniel Kuźnicki, Magdalena Arasimowicz-Jelonek

**Affiliations:** ^1^Department of Plant Ecophysiology, Faculty of Biology, Adam Mickiewicz University in Poznań, Poznań, Poland; ^2^Department of Plant Physiology, Poznań University of Life Sciences, Poznań, Poland

**Keywords:** peroxynitrite, reactive nitrogen species, hypersensitive response, nucleic acid nitration, *Phytophthora infestans*, potato

## Abstract

Peroxynitrite (ONOO^-^) exhibits a well-documented nitration activity in relation to proteins and lipids; however, the interaction of ONOO^-^ with nucleic acids remains unknown in plants. The study uncovers RNA and mRNA nitration as an integral event in plant metabolism intensified during immune response. Using potato-*avr/vr Phytophthora infestans* systems and immunoassays we documented that potato immunity is accompanied by two waves of boosted ONOO^-^ formation affecting guanine nucleotides embedded in RNA/mRNA and protein tyrosine residues. The early ONOO^-^ generation was orchestrated with an elevated level of protein nitration and a huge accumulation of 8-nitroguanine (8-NO_2_-G) in RNA and mRNA pools confirmed as a biomarker of nucleic acid nitration. Importantly, potato cells lacking ONOO^-^ due to scavenger treatment and attacked by the *avr* pathogen exhibited a low level of 8-NO_2_-G in the mRNA pool correlated with reduced symptoms of programmed cell death (PCD). The second burst of ONOO^-^ coincided both with an enhanced level of tyrosine-nitrated proteins identified as subtilisine-like proteases and diminished protease activity in cells surrounding the PCD zone. Nitration of both RNA/mRNA and proteins *via* NO/ONOO^-^ may constitute a new metabolic switch in redox regulation of PCD, potentially limiting its range in potato immunity to *avr P. infestans*.

## Introduction

Peroxynitrite (ONOO^-^) is a product of the extremely rapid and diffusion-controlled reaction between the two radicals, nitric oxide (NO) and superoxide anion (${\text{O}}_{2}^{•-}$). The formation of the NO cognate has been detected *in vivo* in different cellular compartments in plants exposed to various stress factors (e.g., Saito et al., 2006; Corpas et al., 2009; Arasimowicz-Jelonek et al., 2011, 2012; Corpas and Barroso, 2014). However, unlike in animals, ONOO^-^, does not appeared to be a such destructive to plant cell metabolism and is not an essential intermediate of plant cell death (Delledonne et al., 2001; Romero-Puertas et al., 2007). Moreover, it has also been revealed that incubation of *Arabidopsis* plants in a high concentration of ONOO^-^ (3 mM) did not lead to the cell death, even in the Prx II E mutant line with the defective expression of *PrxII E* exhibiting peroxynitrite reductase activity (Romero-Puertas et al., 2007). Additionally, there are also several premises suggesting that ONOO^-^ production occurs in plants as an integral event of cellular metabolism, which is exactly adopted to its accumulation (Romero-Puertas et al., 2004; Gzyl et al., 2016). Therefore ONOO^-^ production could provide an important regulatory loop for NO bioactivity under both physiological and pathophysiological states, since ONOO^-^ can provoke tyrosine nitration, recently considered to be a regulatory mechanism for protein activity (Gzyl et al., 2016).

It is well documented that ONOO^-^ is one of the most important species engaged in nitration of various biomolecules. Apart from proteins it can modify lipids and oligonucleotides, significantly affecting their biochemistry (Jones, 2012). In plants, more than 100 protein involved in a wide range of biological processes in *Arabidopsis thaliana* and other model plants are known to be potential targets for tyrosine nitration, although the functional significance of this modification so far has been proven only for 13 proteins (Chaki et al., 2009, 2013; Álvarez et al., 2011; Galetskiy et al., 2011; Lozano-Juste et al., 2011; Melo et al., 2011; Begara-Morales et al., 2013, 2014, 2015; Corpas et al., 2013; Holzmeister et al., 2015; Sainz et al., 2015; Takahashi et al., 2015). The presence of endogenous nitro-linolenic acid in *Arabidopsis thaliana* was also evidenced, including data supporting the signaling role of these molecules in the tolerance mechanism against different abiotic stress factors such as wounding, salinity, cadmium, and low temperature (Mata-Pérez et al., 2016). The interaction of peroxynitrite with other important biomolecules, such as nucleic acid, to date has not been studied in plant systems. However, it is well established that various RNS, including ONOO^-^ or nitrogen oxides, can nitrate guanine and related nucleosides and nucleotides either in the free form or embedded in DNA and/or RNA (Ihara et al., 2011). Notably, when peroxynitrite react with guanine, it forms several products, among which 8-oxoguanine (8-Oxy-G) and 8-nitroguanine (8-NO_2_-G) are most abundant. Importantly, ONOO^-^ reacts with the DNA and RNA bases at selected positions (Jena and Mishra, 2007). As indicated by Sodum and Fiala (2001), ONOO^-^ can mediate oxidation and nitration of guanine which occurs mainly at the C8 position.

Potato response to the oomycete pathogen *Phytophthora infestans* (Mont.) de Bary is accompanied by changes in the NO metabolic status within the attacked cell (Kato et al., 2013; Abramowski et al., 2015; Arasimowicz-Jelonek et al., 2016; Floryszak-Wieczorek et al., 2016). These include ONOO^-^ formation evidenced as an important redox regulator of defense responses involved in basal resistance (Arasimowicz-Jelonek et al., 2016). Furthermore, selective nitration of tyrosine residues in a small number of proteins recorded during the late phase of resistant response allowed us to form hypothesis that peroxynitrite might act as redox regulator also in cells undergoing hypersensitive cell death. Exploring the functional role of ONOO^-^ in the potato – *P. infestans* pathosystem, the present study demonstrates the first experimental evidence of nucleic acid nitration in plants, since the elevated formation of 8-NO_2_-G within the RNA and mRNA pools was found in response to pathogen attack. What is more, the early ONOO^-^-mediated modifications at mRNA and protein levels favor active cell death and limit its range in potato immunity to *avr P. infestans*.

## Materials and Methods

### Plant Growth

The experiments were conducted on two sterile potato cultivars (*Solanum tuberosum* L.) – cv. Bintje (lacking R genes) which is highly susceptible to isolate 1.3.4.7.10.11. *P. infestans*, and cv. Bzura [carrying the R1 gene (Gebhardt et al., 2004) and the R2-like gene located in chromosome IV (Plich et al., 2015)] – highly resistant to 1.3.4.7.10.11. *P. infestans*. Plants of both cultivars derived from *in vitro* tissue culture were transferred to soil and grown for 4 weeks in a growth chamber with 16 h of light (180 μmol m^-2^ s^-1^) at 18 ± 2°C and 60% humidity.

### Pathogen Culture

*Phytophthora infestans* – isolate 1.3.4.7.10.11. was kindly obtained from the Plant Breeding and Acclimatization Institute, Research Division at Młochów, Poland. The oomycete was grown on a cereal-potato medium with an addition of dextrose.

### Method of Inoculation

For *P. infestans* inoculation, the abaxial site of the detached leaves of both potato cultivars were sprayed with a zoospore suspension in water (conc. 2.0 × 10^5^ per ml) and kept at 100% humidity in a growth chamber. The material for further analysis was taken until 96 h post inoculation (hpi). For the point inoculation experiment, 20 μl of the zoospore suspension were drop inoculated on the abaxial leaf surface and kept at 100% humidity in a growth chamber. The material for analysis was taken at the site of inoculation from an area of 0.5 cm in diameter (PCD zone, 1) and from the surrounding area within the radius 0.25 cm; (PCD distal zone, 2) until 96 hpi.

### Peroxynitrite Donor and Scavenger Treatment

To estimate the effect of exogenous ONOO^-^ detached potato leaves were sprayed with ONOO^-^ donor – 50 μM SIN-1 (3-Morpholinosydnonimine, Calbiochem) which gradually decomposed to yield equimolar amounts of NO and ${\text{O}}_{2}^{•-}$. Scavengers of ONOO^-^ (50 μM ebselen or 1 mM epicatechin, Sigma) were used to evaluate the effect of endogenous on 8-NO_2_-G formation. Control plants were treated with water. After 5 h of incubation leaves were gently dried and inoculated as described above.

### Peroxynitrite Detection

Peroxynitrite formation was measured quantitatively using aminophenyl fluorescein (APF). Leaf disks (0.5 g) were incubated in darkness for 1 h in a mixture containing 5 μM APF in 100 mM phosphate buffer (pH 7.4). After the incubation, the probes were transferred into 24-well plates (1 ml per well) and the fluorescence was measured using spectrofluorometer at 485 nm excitation and 510 nm emission filters. Fluorescence was expressed as a relative fluorescence units.

### TUNEL Assay

The TUNEL assay measures DNA fragmentation using the terminal deoxynucleotidyl transferase (TdT)-mediated deoxyuridine triphosphate (dUTP) nick end labeling method, which involves the TdT-mediated addition of fluorescein-12-dUTP to the 3′-OH ends of fragmented DNA. The samples were studied using TUNEL fluorescein kit (Roche; United States) according to Floryszak-Wieczorek and Arasimowicz-Jelonek (2016) and examined using a fluorescence microscope (Axiostar plus, Carl Zeiss, Germany) equipped with a digital camera, with excitation at 488 nm and emission at 515 nm. Experiments were repeated four times with ten slides per treatment. A region of 100 cells from at least 5 randomly selected slices in each treatment was counted and statistically analyzed.

### RNA Extraction and Poly(A)-RNA Purification

Potato leaves were frozen in liquid nitrogen and stored at -80°C until use. For RNA extraction leaves (0.5 g) were ground to a fine powder in liquid nitrogen, and total RNA was extracted using TriReagent (Sigma) according to the manufacturer’s instructions. The Poly(A)-RNA from previously prepared total RNA was prepared using the GenElute mRNA Miniprep Kit (Sigma Aldrich) according to the manufacturer’s protocol. Briefly, the obtained total RNA was mixed with the binding buffer and oligo(dT) beads followed by 3-min incubation at 70°C and 10-min incubation at room temperature. After centrifugation the pellet of the oligo(dT) polystyrene beads: the mRNA complex was mixed with washing buffer and transferred to spin columns. After the second washing the mRNA was eluted at 70°C using an elution buffer (10 mM Tris-HCl, pH 7.5). The quantity and quality of obtained mRNA were measured by spectrophotometric methods.

### 8-NO_2_-G Quantification

The level of 8-nitroguanine was determined using a competitive enzyme immunoassay OxiSelect^TM^ Nitrosative DNA/RNA Damage ELISA Kit (Cell Biolabs; STA-825), similar to Phookphan et al. (2017). For the analysis, 10 mg of the sample (total RNA or mRNA) was used. The unknown 8-NO_2_-Gua samples or 8-NO_2_-Gua standards were first added to an 8-NO_2_-Gua-BSA conjugate preabsorbed microplate. After a brief incubation, an anti-8-NO_2_-Gua monoclonal antibody was added, which bind to either the 8-nitroguanine of the samples or standards or to the 8-NO_2_-Gua-BSA conjugate preabsorbed on the plate. The more 8-nitroguanine in the sample, the less free antibody available to bind to the conjugate on the plate. The absorbance of the samples was measured at a wavelength of 450 nm using an iMark microplate reader (BioRad). The 8-NO_2_-G content was determined by comparing with the predetermined 8-NO_2_-G standard curve. Each sample was analyzed in triplicate on ELISA microplates and values presented are means of three biological replicates (*n* = 9) ± SD.

### Protein 3-Nitrotyrosine Assay

3-nitrotyrosine in a protein sample was measured using the OxiSelect^TM^ Nitrotyrosine ELISA Kit (Cell Biolabs; STA-305) according to the manufacturer’s protocol. Briefly, nitrated BSA or samples were added to each well of 96-well plates and incubated with an anti-nitrotyrosine antibody at a 1:1000 dilution for 1 h on an orbital shaker. Wells were then washed three times, afterward the secondary antibody was added and the mixture incubated for 1 h at room temperature. Subsequently, the wells were washed three times and the substrate solution was added to each well. Finally after the color development the reaction was stopped and optical density was measured at 450 nm using an iMark microplate reader (BioRad). The 3-nitrotyrosine content in protein samples was determined by comparing with the predetermined 3-nitrotyrosine standard curve. Each sample was analyzed in triplicate on ELISA microplates and the values presented are means of three biological replicates (*n* = 9) ± SD.

### Protease Activity Assay

Protease activity was determined according to Fernández et al. (2015) using azocasein as substrate in 50 mM sodium acetate buffer (pH 5.2) and 0.5 μg of isolated protease. Samples were incubated at 37°C for 2.5 h. Protease activity was measured as an increase in the absorbance at 335 nm of the supernatant.

### Trypan Blue Staining

To visualize cell death the trypan blue dye was used according to Fernández-Bautista et al. (2016).

### Statistical Analysis

All results are based on three biological replicates derived from three or four independent experiments. For each experiment, means of the obtained values were calculated along with standard deviations. The analysis of variance was performed (ANOVA) and the mean values were compared by Tukey’s test (α = 0.05).

## Results

### *P. infestans* Provokes Two Waves of ONOO^-^ Formation During Potato Immune Response

**FIGURE 1:**
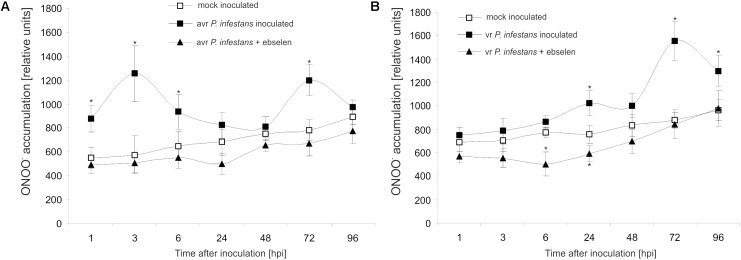
Peroxynitrite formation measured as APF fluorescence in leaves of resistant **(A)** and susceptible **(B)** potato inoculated with *P. infestans*. Analyses were performed at 1, 3, 6, 24, 48, 72, and 96 h after challenge inoculation. Values represent the mean ± SD of at least three independent experiments (*n* = 9). Asterisks indicate values that differ significantly from mock inoculated (control) potato leaves at *P* < 0.05 (^∗^).

Based on the folic acid method, we previously reported that the *P. infestans* challenge provoked an early and transient program of boosted ONOO^-^ formation only in the resistant potato genotype (Arasimowicz-Jelonek et al., 2016). In the present study the ONOO^-^ production in potato leaves was detected quantitatively using APF fluorochrome up to 96 hpi. Bio-monitoring confirmed that the potato–*avr P. infestans* interaction is accompanied by a significant ONOO^-^ accumulation within the first 6 hpi; however, monitoring within the successive hours revealed a second, lower burst of ONOO^-^ at 72 hpi (**Figure 1A**). The potato–*vr P. infestans* pathosystem revealed ONOO^-^ formation starting from 24 hpi, which reached the highest (*ca.* 2-fold increase) level at 72 hpi (**Figure 1B**).

Moreover, to verify that the detected pathogen-induced increase in APF fluorescence was caused by endogenous ONOO^-^, its detection was performed in the presence of ebselen, an ONOO^-^ scavenger. The used scavenger strongly suppressed fluorescence at 3rd and 24th hpi (**Figures 1A,B**), providing evidence that APF is a reliable tool to quantitatively investigate ONOO^-^ formation in potato leaves.

### The Presence of ONOO^-^ Is Necessary for PCD During HR

**FIGURE 2:**
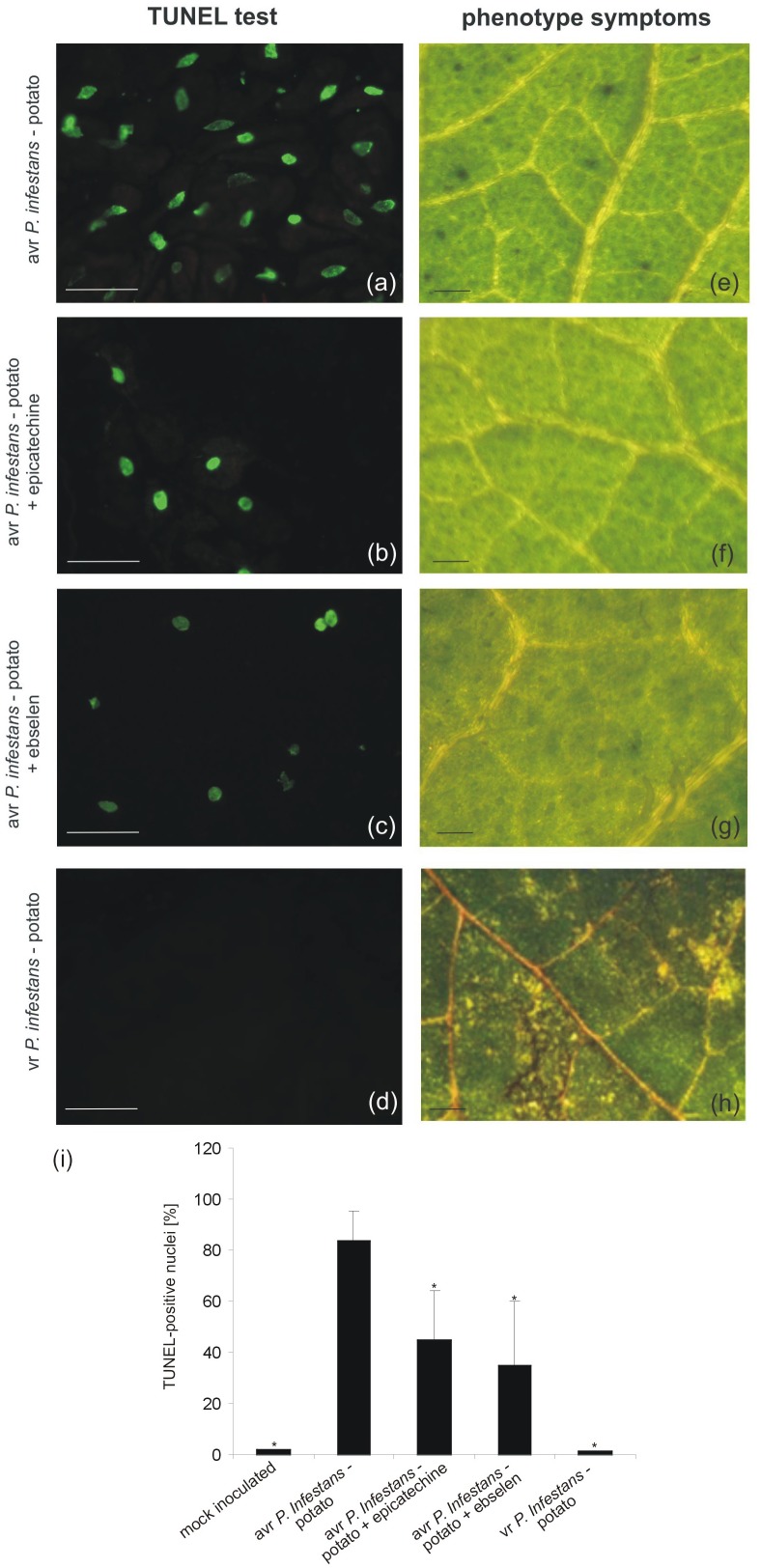
TUNEL test illustrating programmed DNA fragmentation **(a–d)** and phenotype symptoms **(e–h)** on potato leaves inoculated with *P. infestans* at 48 hpi. Representative images of TUNEL-positive nuclei **(a)** and HR-like lesions **(e)** observed during the *avr P. infestans*-potato interaction; TUNEL-positive reaction in leaves of potato pretreated with epicatechin and next inoculated with *avr P. infestans* **(b)**; TUNEL-positive reaction in leaves of potato pretreated with ebselen and next inoculated with *avr P. infestans* **(c)**; TUNEL-negative reaction **(d)** and disease spots **(h)** observed during the *vr P. infestans*-potato interaction. Bars indicate 15 μm **(a–d)** and 500 μm **(e–h)**. The percentage of leaf cells exhibiting the TUNEL-positive reaction at 48 hpi **(i)**, 100 cells from at least 5 randomly selected slides were examined at each time point per treatment. Values represent the mean ± SD of four independent experiments (*n* = 20). Asterisks indicate values that differ significantly from *P. infestans* inoculated potato leaves at *P* < 0.05 (^∗^).

The potato–*avr P. infestans* interaction resulted in HR-like cell death (Floryszak-Wieczorek and Arasimowicz-Jelonek, 2016). To gain insight into ONOO^-^ participation in the death of pathogen attacked cells, the TUNEL assay illustrating the programmed DNA fragmentation in the presence of ONOO^-^ scavengers was performed (**Figure 2**). Cells of potato leaves treated with *avr P. infestans* contained green-colored nuclei starting from 24 hpi (**Supplementary Figure S1**), while phenotype HR-like symptoms appeared 48 h after the *avr P. infestans* challenge (**Figure 2e**). The number of TUNEL–positive nuclei indicated that the effect was dependent on the ONOO^-^ presence within potato leave cells, since the sequential treatment with epicatechin, a ONOO^-^ scavenger and *P. infestans* effectively reduced the number of cells with symptoms of active death from 85 to 45% at 48 hpi (**Figures 2b,i**). Similarly, potato cells pretreated with ebselen and next elicited by the pathogen at 48 hpi showed 35% of nuclei with TUNEL–positive staining (**Figures 2c,i**). An independent application of both ONOO^-^ scavengers resulted in reduced phenotype HR-like symptoms (**Figures 2f,g**).

### Peroxynitrite Mediates 8-NO_2_-G Formation Within RNA and mRNA Pools During Potato-*P. infestans* Interaction

**FIGURE 3:**
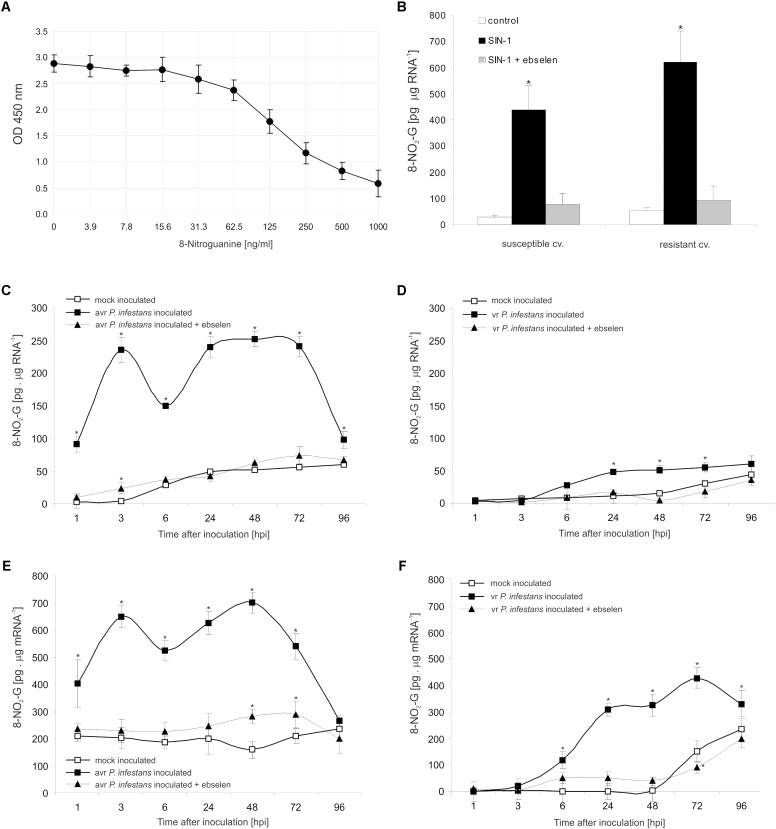
RNA and mRNA nitration in healthy and challenge inoculated leaves of potato. 8-Nitroguanine ELISA standard curve **(A)** in the concentration range of 0 ng/mL to 1000 ng/mL; **(B)** quantification of nitrated RNA measured as 8-NO_2_-G content in resistant and susceptible healthy potato leaves enriched with ONOO^-^; 8-NO_2_-G content was estimated 5 h after leaf pretreatment with 50 μM SIN-1. Quantification of nitrated RNA measured as 8-NO_2_-G content in leaves of resistant **(C)** and susceptible **(D)** potato inoculated with *P. infestans*. Quantification of nitrated mRNA measured as 8-NO_2_-G content in leaves of resistant **(E)** and susceptible **(F)** potato inoculated with *P. infestans*. Analyses were performed at 1, 3, 6, 24, 48, 72, and 96 h after challenge inoculation. Values represent the mean ± SD of at least three independent experiments (*n* = 9). Asterisks indicate values that differ significantly from mock inoculated (control) potato leaves at ^∗^*P* < 0.05.

To explore the functional role of ONOO^-^ generation in potato leaves challenge-inoculated with *P. infestans* we examined whether, and to what extent, this RNS is able to target nucleic acids. Firstly, we confirmed that SIN-1 is effective in ONOO^-^ formation within potato cells (**Supplementary Figure S2**). Secondly, to verify that ONOO^-^ is able to provoke nitration at the RNA level in cells of potato, we monitored the accumulation of 8-NO_2_-G, a marker of nucleic acid nitration, in healthy leaves treated with the ONOO^-^ generator. As we expected, SIN-1 was effective in 8-NO_2_-G RNA formation within potato cells of both genotypes (**Figures 3A,B**).

The potato–*avr P. infestans* interaction resulted in an impressive rise in the level of 8-NO_2_-G within the RNA pool starting from the 1st hpi (**Figure 3C**). The highest increase of nitrated RNA in relation to mock inoculated leaves was noted at 3 hpi; however, a strong, *ca.* 5-fold enhancement of 8-NO_2_-G RNA was observed also between 24 and 72 hpi. Then the level of 8-NO_2_-G RNA declined. Surprisingly, RNA from cells of the susceptible genotype revealed a time-delayed and definitely lower level of 8-NO_2_-G than did RNA of the resistant potato (**Figure 3D**). Only a 2-fold increase in RNA nitration was observed starting from 24 hpi and the enhancement was maintained during disease progress.

Since mRNA modifications have the potential to affect most post-transcriptional steps in gene expression (Gilbert et al., 2016), in the next set of experiments we verified if ONOO^-^ targets mRNA in potato leaves as well. Interestingly, the use of poly(A)-RNA purified from total RNA showed a significant 2-fold increase in the level of 8-NO_2_-G mRNA at 1 hpi, peaking at 3 and 48 hpi, respectively (**Figure 3E**). The amount of 8-NO_2_-G mRNA in cells of the susceptible genotype was raised at 6 hpi, reaching the highest level at 72 hpi (**Figure 3F**). It should be highlighted that the steady-state level of 8-NO_2_-G mRNA observed within the first 48 h of the experiment was significantly higher in healthy leaves of resistant potato than in the susceptible one (**Figures 3E,F**). What is more, cells of both potato genotypes lacking ONOO^-^ due to the scavenger application and next attacked by the pathogen exhibited significantly lower levels of 8-NO_2_-G both in RNA and mRNA (**Figures 3C–F**).

### Protein Nitration as a Switch of the Redox Environment During HR Establishment

**FIGURE 4:**
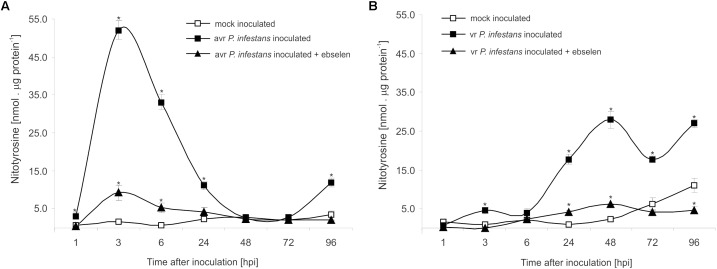
Quantification of protein tyrosine nitration in leaves of resistant **(A)** and susceptible **(B)** potato inoculated with *P. infestans*. Analyses were performed at 1, 3, 6, 24, 48, 72, and 96 h after challenge inoculation. Values represent the mean ± SD of at least three independent experiments (*n* = 9). Asterisks indicate values that differ significantly from mock inoculated (control) potato leaves at ^∗^*P* < 0.05.

**FIGURE 5:**
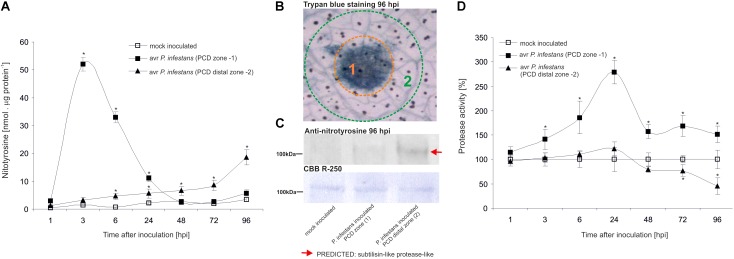
Quantification of protein tyrosine nitration **(A)** and protease activity **(D)** in leaves of resistant potato at the site of *avr P. infestans* inoculation (PCD zone) and in the surrounding area (PCD distal zone). Analyses were performed at 1, 3, 6, 24, 48, 72, and 96 h after challenge inoculation. Values represent the mean ± SD of at least three independent experiments (*n* = 9). Asterisks indicate values that differ significantly from mock inoculated (control) potato leaves at ^∗^*P* < 0.05. **(B)** Trypan blue staining illustrating the PCD zone (1) and PCD distal zone (2) at 96 hpi. **(C)** Representative immunoblot showing immunoreactive bands containing subtilisin-like proteins undergoing tyrosine nitration in potato leaves challenge inoculated with *avr P. infestans* at 96 hpi. Western blot was probed with a rabbit anti-nitrotyrosine polyclonal antibody at a 1:1.000 dilution. The loading control was determined by staining the blot with CBB R-250. The indicated band was isolated from PVDF membrane and identified by LC-MS-MS/MS as described in **Supplementary Data Sheet S1**.

The nitrative modification of RNA and mRNA overlapped with the tyrosine residue nitration in proteins within the first 24 h after *avr P. infestans* challenge inoculation (**Figure 4A**). Based on the immunoassay (**Supplementary Figure S3**) we found the highest, *ca.* 30-fold increase in the total protein pool undergoing tyrosine nitration at 3 hpi (**Figure 4A**). Then, the level of nitrated proteins was gradually decreased and reached the amount recorded in mock inoculated leaves at 48 and 72 hpi. Interestingly, a *ca.* 3-fold increase of nitrated proteins at 96 hpi was followed by the second burst of ONOO^-^ formation (**Figure 4A**). Additional experiment involving point inoculation revealed that only cells surrounding the PCD zone showed an elevated level of protein nitration at 96 hpi identified as subtilisin-like proteases (**Figures 5A–C** and **Supplementary Tables S1**,**S2**). Moreover, changes noted within the PCD distal zone coincided with the significantly reduced protease activity (**Figure 5D**). In contrast, disease progress observed during the potato-*vr P. infestans* interaction was accompanied by an increase of the nitrated protein pool starting from 24 hpi (**Figure 4B**).

## Discussion

The hypersensitive response (HR) considered as a form of programmed cell death (PCD) is a hallmark of effector-triggered immunity (ETI). According to current knowledge, properly balanced doses of NO and ROS co-operate to trigger PCD in plants (Delledonne et al., 2001; Zago et al., 2006). To date the role of ONOO^-^ in HR has been ambiguous. Although application of 1 mM ONOO^-^ has been reported to cause some necrotic lesions in *Arabidopsis* (Alamillo and García-Olmedo, 2001), high concentration of peroxynitrite does not seem to be essential to PCD induction (Delledonne et al., 2001; Romero-Puertas et al., 2007). That is probably due to the capacity of plants to detoxify ONOO^-^ under normal physiological conditions.

Some evidence demonstrated that ONOO^-^ in plants fulfills an important role in the creation of a cellular redox milieu promoting defense expression (Durner et al., 1998; Alamillo and García-Olmedo, 2001; Arasimowicz-Jelonek et al., 2016). Our earlier results showed that NO and O_2_^-^ coexisted after potato leaf inoculation (Arasimowicz-Jelonek et al., 2016) and both molecules were needed to activate PCD in the potato-*avr P. infestans* system. Using the spectrofluorometric method for ONOO^-^ detection we verified that the potato-*avr P. infestans* interaction is accompanied by ONOO^-^ formation within the first 6 hpi and revealed an additional wave of ONOO^-^ during the following hpi. The first burst of ONOO^-^ definitely preceded the TUNEL-positive reaction of cell nuclei. What is more, significantly reduced numbers of cells undergoing PCD were observed in potato leaves pretreated with epicatechin and ebselen, confirming that ONOO^-^ next to NO and ROS participates in the active cell-death program.

Searching for a novel link connecting an early ONOO^-^ formation with PCD during the potato-*avr P. infestans* interaction we found that not only protein tyrosine residues are affected by nitration activity of this RNS. In this study we demonstrated for the first time that also the guanine nitration phenomenon occurs in plant cells. Nitration of nucleotides in DNA and RNA and the resulting 8-NO_2_-G formed in response to RNS were first suggested to be activated in hamster livers infected with *Opisthorchis viverrini* (Pinlaor et al., 2003), and in the human gastric mucosa infected with *Helicobacter pylori* (Ma et al., 2004). In both cases the phenomenon was associated with infection- or inflammation-induced carcinogenesis (Terasaki et al., 2006). Although most of the studies regarding to nucleic acid nitration is focused on DNA, several reports indicate that RNA is more susceptible to this phenomenon (Shan et al., 2007; Liu et al., 2012; Hawkins et al., 2017).

8-NO_2_-G was detected in both potato genotypes challenge inoculated with the pathogen. However, only the resistant response was accompanied by a significant accumulation of the nitrated RNA pool starting from the first hpi. What is more, the modification dropped after 72 hpi. A similar trend was observed also within the mRNA pool. Thus, we conclude that mRNA nitration is an early event preceding or coincident with the first symptoms of PCD during HR and this process is not merely a consequence of dying cells. In confirmation, disease symptom development was accompanied by low levels of RNA/mRNA nitration starting from 24 hpi and correlated with necrotic cell dying. Formation of 8-NO_2_-G in RNA may interfere with RNA functions and metabolism, similarly as RNA modification *via* ROS (Kong and Lin, 2011; Wang and He, 2014; Fimognari, 2015). It was earlier suggested that mRNA may be a major target of oxidative modification because of its relative abundance, widespread subcellular distribution, single-strand nature and, lack of protection from histone proteins (Shan et al., 2007). Messenger RNA modification *via* oxidation was found to be an early event prior to cell death in animals, rather than a simple consequence of an already dying cell, and it was shown to induce reduced protein expression (Shan et al., 2007). The effect of oxidized bases in mRNAs may cause ribosome stalling on the transcripts, leading to a decrease of protein expression or slowing the translation process. In a similar manner, an important consequence of RNA nitration could be an impairment of protein synthesis (Rivera-Mancía et al., 2017). Briefly, targeted RNA nitration might lead to the diminished expression of specified proteins and thus constitute a mechanism of post-transcriptional gene expression regulation. What is more, oxidized transcripts could be subjected to ribosome-based quality control and predestined for degradation through No-Go decay pathway (Simms et al., 2014). It should be noted that targeted mRNA oxidation has also been documented in plants. Namely, increased levels of 8-oxo-7,8-dihydroguanosine (8-OHG), which is a marker of RNA oxidation, were detected in sunflower seeds. Importantly, the observed modification was not a random process, but highly selective, directed toward a specific subset of 24 mRNAs, including mRNAs corresponding to genes associated with cell signaling (Bazin et al., 2011). So far, it is not clear what determine the susceptibility of mRNA to this type of modification. However, it has been proved that its sensitivity is not dependent on the abundance of specific mRNA species in the cell, the frequency of guanine in the sequence or the occurrence of any specific motif (Chang et al., 2008; Bazin et al., 2011). It should be noted that the observed increase in 8-NO_2_-G mRNA in healthy leaves of susceptible potato might hinder protein synthesis or generate errors in the protein products related to senescence disorders.

**FIGURE 6:**
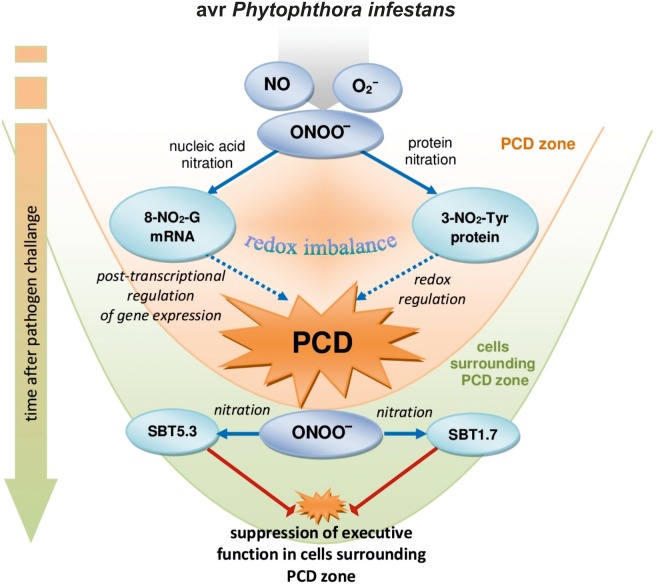
A functional link between peroxynitrite and potato immunity in response to *avr P. infestans*: the early ONOO^-^ generation facilitates nitrative modification of nucleic acids that could contribute to the post-transcriptional regulation of gene expression unlocking programmed cell death during HR; the second burst of ONOO^-^ could favor protease inactivation *via* protein nitration suppressing the potential executer function of subtilisin-like proteases in cells distal from the zone of active dying cells.

Nitration of both guanine nucleotides embedded in RNA and mRNA and protein tyrosine residues can constitute an early switch of the redox environment facilitating HR establishment. Cecconi et al. (2009) observed *in vivo* an increase in nitrated proteins during the progression of hypersensitive response. In *A. thaliana* challenge inoculated with an avirulent bacterial pathogen defense responses were correlated with a modulation of nitrated proteins involved in regulation of a number of important cellular functions after 4 and 8 hpi confirming that Tyr-nitration could be a relevant physiological process in resistance (Gaupels et al., 2011). More recently, we found that *avr P. infestans* in potato leaves provoked nitration of subtilisin-like proteases, i.e., SBT1.7 and SBT5.3 belonging to serine-dependent enzymes at 48 hpi (Arasimowicz-Jelonek et al., 2016). Importantly, subtilisin-like protease (StSBTc-3) induced in potato leaves after *P. infestans* infection was found to exhibit caspase-3 like activity and display an executer function (Fernández et al., 2015). Since a physiological consequence of the ONOO^-^ reaction with proteins often involves inactivation or impairment of its function, therefore nitration following the second burst of ONOO^-^ may efficiently inhibit the activity of serine proteases and suppress a potential executer function in the distal zone from dying cells (**Figure 6**). In confirmation, an experiment applying point inoculation revealed nitration of SBT1.7 and SBT5.3 concomitant with reduced protease activity only in cells surrounding the PCD zone. It should be noted that the distal changes were not correlated with nitrative mRNA modification (data not presented).

## Conclusion

Our study demonstrates that the nitration phenomenon presents a much more complex functionality in plant cells than it was assumed previously. The modification of RNA and mRNA *via* ONOO^-^ is an integral part of plant cell metabolism and is intensified in response to pathogen attack. Although nitrative modification of bases in RNA and mRNA can be simply induced by an enhanced peroxynitrite level in the cellular milieu, the rate of ONOO^-^ formation is dependent on the plant genetic makeup. An early and transient program of boosted ONOO^-^ formation during the potato resistant response accelerated the time-dependent switch of the redox environment *via* the nitration phenomenon. The observed nitrative modification of RNA and mRNA could regulate the post-transcriptional gene expression and fine-tune cell signaling that contributes to PCD during HR (**Figure 6**). In confirmation, ONOO^-^ elimination overlapped with a reduced pool of nitrated mRNA and the number of cells that undergo programmed cell death. In contrast, a time-delayed peroxynitrite over-accumulation in the potato-*vr P. infestans* interaction coincident with a relatively low level of 8-NO_2_-G in the RNA/mRNA pools resulted in failed resistance. The challenge for the future is to understand the mechanisms of selective mRNA nitration and the physiological consequences of mRNA nitration. To this aim identification of mRNA nitration targets during plant responses to pathogen attack should be experimentally verified.

## Author Contributions

MA-J and JF-W planned and designed the research. KI, JG, BM, and DK performed the experiments. KI collected, analyzed the data and participated in writing the manuscript. MA-J wrote the manuscript.

## Conflict of Interest Statement

The authors declare that the research was conducted in the absence of any commercial or financial relationships that could be construed as a potential conflict of interest.

## Supplementary Material

FIGURE S1 Percentages of leaf cells exhibiting the TUNEL-positive reaction at 24, 48, and 72 hpi (i), 100 cells from at least 5 randomly selected slides were examined at each time point per treatment. Values represent the mean ± SD of at least four independent experiments (*n* = 20). Asterisks indicate values that differ significantly from *P. infestans* inoculated potato leaves at ^∗^*P* < 0.05.

FIGURE S2 ONOO^-^ generation measured as APF fluorescence in healthy leaves of resistant and susceptible potato in response to increasing SIN-1 concentration. Values represent the mean ± SD of at least three independent experiments (*n* = 9). Asterisks indicate values that differ significantly from non-treated potato leaves at ^∗^*P* < 0.05.

FIGURE S3 **(a)** 3-Nitrotyrosine ELISA standard curve in the concentration range of 0–8000 nM; **(b)** quantification of nitrated proteins measured as 3-nitrotyrosine content in resistant and susceptible healthy potato leaves enriched with ONOO^-^; 3-nitrotyrosine content was estimated 5 h after leaf pretreatment with 50 μM SIN-1.

TABLE S1 Identification of nitrated proteins in potato leaves inoculated with *avr P. infestans* which appeared de novo at 96 hpi.

TABLE S2 Amino acid sequence of the identified homologous proteins (matched peptides derived from *Solanum tuberosum* shown in Bold Red).

DATA SHEET S1 Nitrotyrosine immunodetection and protein identification procedures.
