# Supplementary figures and images for: RNA and mRNA Nitration as a Novel Metabolic Link in Potato Immune Response to Phytophthora infestans
Source: Front Plant Sci. 2018 May 29;9:672. doi: 10.3389/fpls.2018.00672 (PMC5987678; doi:10.3389/fpls.2018.00672)

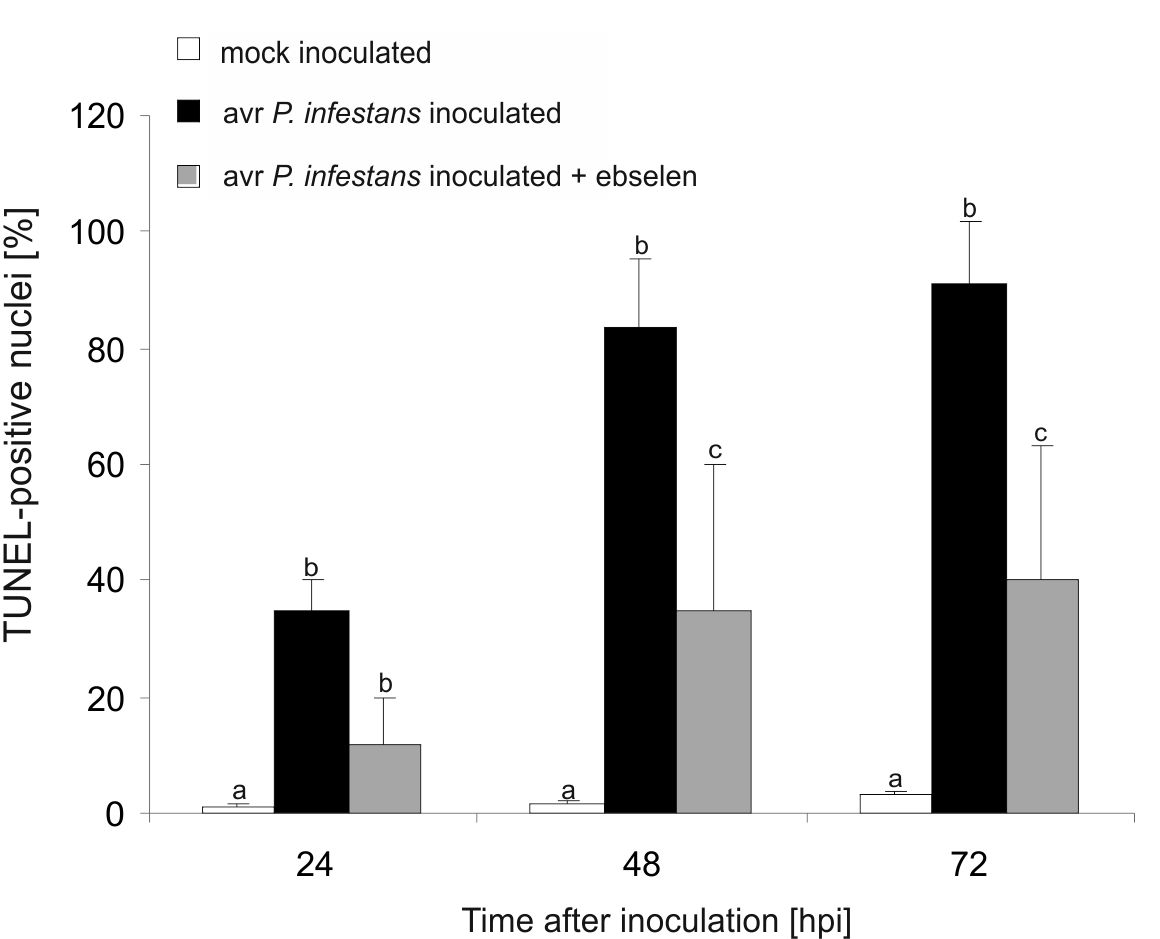

Supplement: FIGURE S1 — Percentages of leaf cells exhibiting the TUNEL-positive reaction at 24, 48, and 72 hpi (i), 100 cells from at least 5 randomly selected slides were examined at each time point per treatment. Values represent the mean ± SD of at least four independent experiments (n = 20). Asterisks indicate values that differ significantly from P. infestans inoculated potato leaves at ∗P < 0.05. [file Image_1.JPEG]

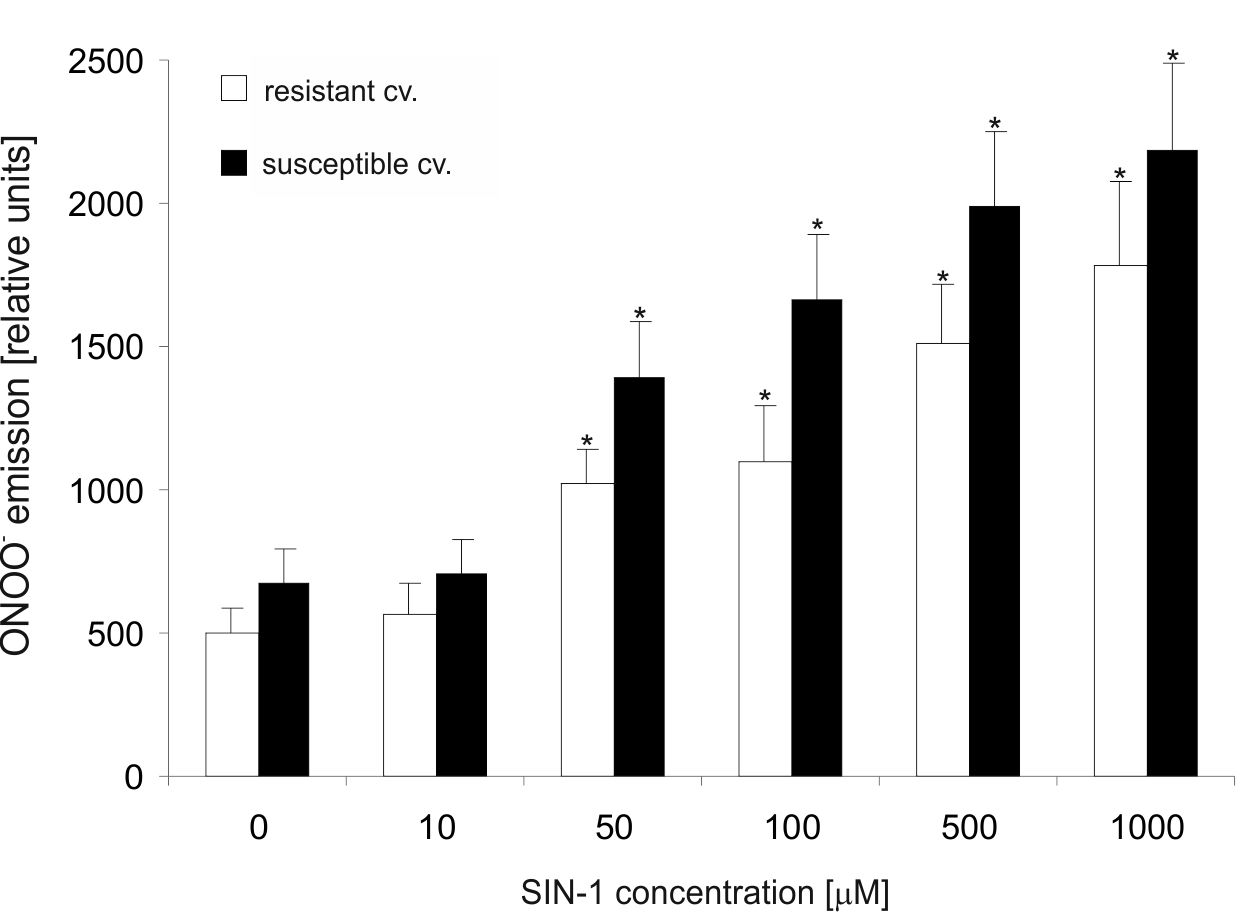

Supplement: FIGURE S2 — ONOO- generation measured as APF fluorescence in healthy leaves of resistant and susceptible potato in response to increasing SIN-1 concentration. Values represent the mean ± SD of at least three independent experiments (n = 9). Asterisks indicate values that differ significantly from non-treated potato leaves at ∗P < 0.05. [file Image_2.JPEG]

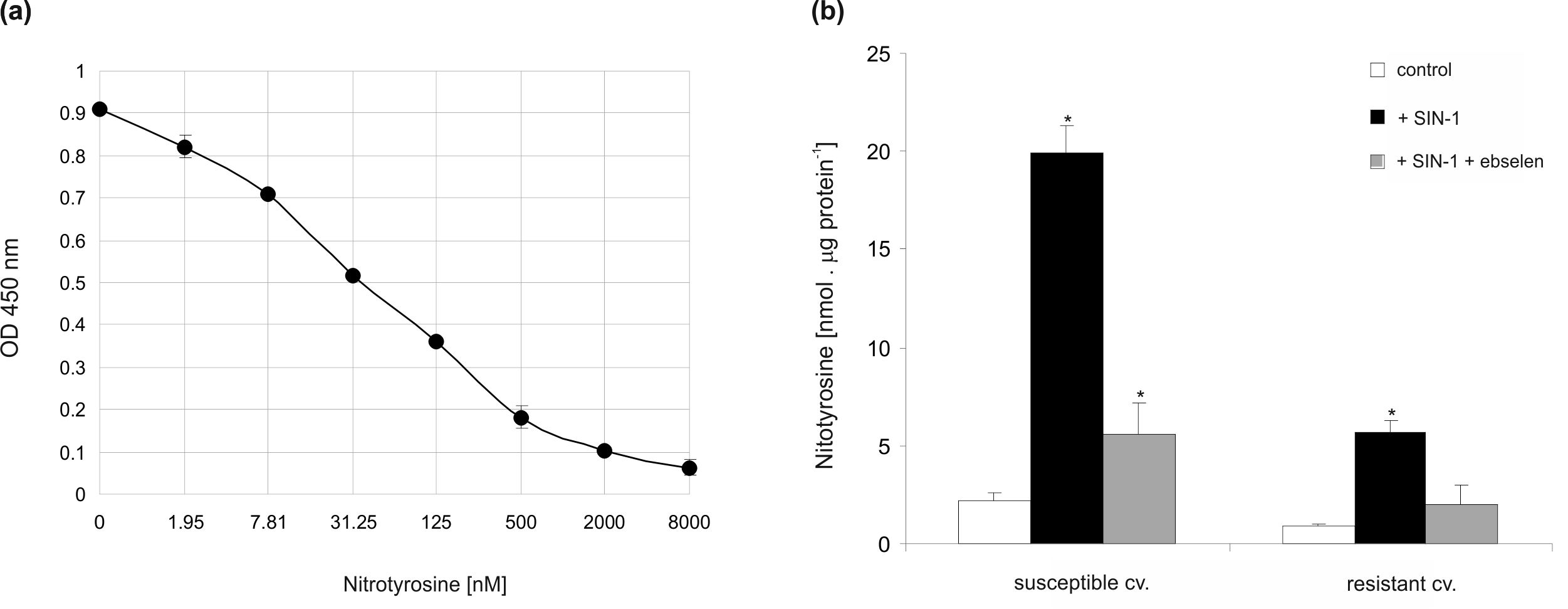

Supplement: FIGURE S3 — (a) 3-Nitrotyrosine ELISA standard curve in the concentration range of 0–8000 nM; (b) quantification of nitrated proteins measured as 3-nitrotyrosine content in resistant and susceptible healthy potato leaves enriched with ONOO-; 3-nitrotyrosine content was estimated 5 h after leaf pretreatment with 50 μM SIN-1. [file Image_3.JPEG]
