# Supplementary material for: RNA and mRNA Nitration as a Novel Metabolic Link in Potato Immune Response to Phytophthora infestans
Source: Front Plant Sci. 2018 May 29;9:672. doi: 10.3389/fpls.2018.00672 (PMC5987678; doi:10.3389/fpls.2018.00672)
Supplement: TABLE S1 — Identification of nitrated proteins in potato leaves inoculated with avr P. infestans which appeared de novo at 96 hpi. [file Table_1.DOCX]

| **No.** | **Protein name** | **NCBI**  **accession no.** | **Blast Score** | **Functional category** |
| --- | --- | --- | --- | --- |
| **1** | PREDICTED: subtilisin-like protease-like | gi\|565378238 | 885 | Proteolysis |
|  | PREDICTED: subtilisin-like protease-like | gi\|565378352 | 245 | Proteolysis |

Table S1. Identification of nitrated proteins in potato leaves inoculated with *avr* *P. infestans* which appeared *de novo* at 96 hpi. Proteins were isolated from PVDF membrane and identified by LC-MS-MS/MS.
