# Supplementary material for: RNA and mRNA Nitration as a Novel Metabolic Link in Potato Immune Response to Phytophthora infestans
Source: Front Plant Sci. 2018 May 29;9:672. doi: 10.3389/fpls.2018.00672 (PMC5987678; doi:10.3389/fpls.2018.00672)
Supplement: TABLE S2 — Amino acid sequence of the identified homologous proteins (matched peptides derived from Solanum tuberosum shown in Bold Red). [file Table_2.DOCX]

Table S2. Amino acid sequence of the identified homologous proteins (matched peptides derived from *Solanum tuberosum* shown in **Bold Red**)

| **No.** | **NCBI**  **accession no.** |  |
| --- | --- | --- |
| **1** | gi\|565378238 | \| 1 \| MKNHHIIVFL \| FSFFLIMSLR \| GFVSCTDQET \| KVYVVYLGEH \| NGEKTLKEIE \| \| --- \| --- \| --- \| --- \| --- \| --- \| \| 51 \| DHHYSFLHSV \| KGTTTSKEDV \| RASLVHSYKN \| VINGFSAVLT \| PQEVDMISGM \| \| 101 \| EGVVSVFHSD \| PYEIRPHTTR \| **SWDFVSLLEG** \| **TSLLNSR**EEL \| LQNASYGKDI \| \| 151 \| IVGVMDSGVW \| PESLSFSDEG \| MEPVPKSWNG \| ICQEGVAFNA \| SHCNRKLIGA \| \| 201 \| RYYLKGYEAA \| AGPLNETRDF \| RSPR**DVDGHG** \| **THTAGTVGGR** \| RVANASAIGG \| \| 251 \| FAK**GTATGGA** \| **PNVR**LAIYKV \| CWPAPDQSLA \| EGNICATDDI \| LAAFDDAIAD \| \| 301 \| GVHVLSISLG \| SLPK**STYYTE** \| **NAIAIGSLHA** \| **VK**KNIVVAC**S** \| **AGNDGPTPST** \| \| 351 \| **VANVAPWVIT** \| **VGASTIDR**VF \| SSPIMLGNGM \| IVEGQTITQI \| RRRR**LHPLVY** \| \| 401 \| **AGDVEIR**GTT \| ASNTTGACLP \| GTLSRNLVRG \| KVVLCLNSDI \| QASMEVKRAG \| \| 451 \| GVAAILGNPF \| NEIQVIPFLN \| PTTVTFLDGL \| **NTLLTYIRTE** \| **KHPTATLVPG** \| \| 501 \| **NTMIGTKTAP** \| **VMAPFSSKGP** \| **NVVDPNILKP** \| **DITAPGFNIL** \| **AAWSEASSPL** \| \| 551 \| **NIPEDHR**VVK \| YNIDSGTSMS \| CPHVSAVIAL \| LK**SIHPDWSS** \| **AAVRSALMTT** \| \| 601 \| **STINNVVGRP** \| **IK**NATGDDAN \| PFEYGSGHFR \| PSKAADPGLI \| YDATYTDYLL \| \| 651 \| YLCSQNIRPD \| LSYNCPAK**VP** \| **AASNLNYPSL** \| **AIANMR**GSSK \| TVTRVVTNVG \| \| 701 \| KDNSTYVVAV \| **RSPPGYAVDI** \| **VPK**SLRFSKL \| GEKHSFNITI \| IRAQSSVDRR \| \| 751 \| NEFSFGR**YTW** \| **SDGVHVVQSP** \| **IAVSSSA** \|  \|  \| |
|  | gi\|565378352 | \| 1 \| MRLGSFVSCT \| EETKVYIVYL \| GEHNGDKTLK \| EIEDHHCSFL \| HSVKGTTSKE \| \| --- \| --- \| --- \| --- \| --- \| --- \| \| 51 \| DVRASLVHSY \| KNVINGFSAV \| LTPQEVDMIS \| GMEGVVSVFH \| SDPYEIRPHT \| \| 101 \| TR**SWDFVSLL** \| **EGTSLLNSR**E \| **ELLQNASYGK** \| **DIIVGVMDSG** \| VWPESSSFND \| \| 151 \| EGMEPVPKSW \| NGICQEGVAF \| NSSHCNRKLI \| GARYYLKGYE \| AAAGPLNETR \| \| 201 \| DFRSPR**DVDG** \| **HGTHTAGTVG** \| **GR**RVANASAI \| GGFAK**GTATG** \| **GAPNVR**LAIY \| \| 251 \| KVCWPVPDQS \| LAEGNACATD \| DILAAFDDAI \| ADGVHVLSIS \| LGSLPK**STYY** \| \| 301 \| **TENAIAIGSL** \| **HAVK**KNIVVA \| CSAGNDGPTP \| STVGNVAPWI \| ITVGASSIDR \| \| 351 \| VFSSPIMLGN \| GMIVEGQTVT \| **PIRRRRLHPL** \| **VYAGDVEIR**G \| TTTNNTSGTC \| \| 401 \| LPGTLSRNLV \| RGKVVLCINN \| LRAASMEVKR \| AGGVAAILGN \| RFNEIQVTPF \| \| 451 \| LDTTTVVFSY \| SLNTLLTYIR \| TEKNPMATLV \| PGNTLIGTK**P** \| **APVMASFTSK** \| \| 501 \| GPNIVDPN**IL** \| **KPDITAPGFN** \| **ILAAWSEASS** \| **PLK**MPEDRRV \| **VKYNMQSGTS** \| \| 551 \| **MSCP**HVSAVI \| ALLKSIHPDW \| SSAAIR**SALM** \| **TTSTINNVVG** \| **RPIK**NATGDD \| \| 601 \| ANPFEYGSGH \| FRPSRAVDPG \| LVYDATYTDY \| LLYLCSQNIS \| LDSSFSCPEK \| \| 651 \| VPTASNLNYP \| SLAIANMRGS \| IRTVTRVVTN \| VGKDNSTYVL \| GVRSPPGYVV \| \| 701 \| DIVPKSLHFS \| KLGEKHSFNI \| TIIRAQSSVE \| RRNEFSFGWY \| TWNDGVHVVR \| |
