# Supplementary material for: RNA and mRNA Nitration as a Novel Metabolic Link in Potato Immune Response to Phytophthora infestans
Source: Front Plant Sci. 2018 May 29;9:672. doi: 10.3389/fpls.2018.00672 (PMC5987678; doi:10.3389/fpls.2018.00672)
Supplement: DATA SHEET S1 — Nitrotyrosine immunodetection and protein identification procedures. [file Data_Sheet_1.DOCX]

**Protein extraction, SDS-PAGE and Western blot**

Leaves (0.25 g) were ground in liquid N_2_ to a fine powder and then suspended in a ratio of 1 to 3 (w/v) in 50mM Tris-HCl buffer (pH 7.6) with 2mM EDTA, 4mM DTT, 0.6% PVPP, 1mM PMSF and plant inhibitor cocktail (Sigma). The crude extracts were centrifuged at 10 000g for 15min at 4ºC, then the concentration of supernatant proteins was determined with the Bradford assay, using BSA as a standard. The solution of proteins was mixed with the Laemmli sample buffer and boiled for 10 min at 70ºC. The sample of proteins (50 µg each) was separated on a gradient SDS PAGE (4% - 20%, BioRad) and gels were stained with CBB-G250 or proteins were electroblotted to PVDF membranes. After transfer, membranes were blocked (5% BSA) and used for cross-reactivity assays with polyclonal antibodies against nitrotyrosine (Life Technologies) at a dilution of 1:1 000. For immunodetection, the goat anti-rabbit antibody conjugated to horseradish peroxidase (Agrisera) and Lumi-Light Western Blotting Substrate (Roche) was used. The intensity of bands was quantified using a Gel Doc system (Bio-Rad) coupled with a highly sensitive camera.

**MS and protein identification**

Protein identification was performed using liquid chromatography coupled to the mass spectrometer at the Laboratory of Mass Spectrometry, Institute of Biochemistry and Biophysics, Polish Academy of Sciences (Warsaw, Poland). Raw data files were pre-processed with the Mascot Distiller software (version 2.4.2.0, MatrixScience). The obtained peptide masses and fragmentation spectra were matched to the National Center Biotechnology Information (NCBI) non-redundant database (37425594 sequences; 13257553858 residues), with a *Viridiplantae* filter (1760563 sequences) using the Mascot search engine (Mascot Daemon v. 2.4.0, Mascot Server v. 2.4.1, MatrixScience). The following search parameters were applied: enzyme specificity was set to semiTrypsin, peptide mass tolerance to ± 30 ppm and fragment mass tolerance to ± 0.1 Da. The protein mass was left as unrestricted, and mass values as monoisotopic with two missed cleavage being allowed. Alkylation of cysteine by carbamidomethylation as fixed, oxidation of methionine and carboxymethylation on lysine were set as a variable modification.

Protein identification was performed using the Mascot search engine (MatrixScience), with the probability based algorithm. The expected value threshold of 0.05 was used for analysis, which means that all peptide identifications had less than 1 in 20 chance of being a random match.
